# Parabolic replicator dynamics and the principle of minimum Tsallis information gain

**DOI:** 10.1186/1745-6150-8-19

**Published:** 2013-08-11

**Authors:** Georgy P Karev, Eugene V Koonin

**Affiliations:** 1 National Center for Biotechnology Information, NLM, National Institutes of Health, Bethesda, Maryland 20894, USA

**Keywords:** Replicator equation, Parabolic growth, Tsallis entropy, Non-extensive statistical mechanics, MaxEnt principle

## Abstract

**Background:**

Non-linear, parabolic (sub-exponential) and hyperbolic (super-exponential) models of prebiological evolution of molecular replicators have been proposed and extensively studied. The parabolic models appear to be the most realistic approximations of real-life replicator systems due primarily to product inhibition. Unlike the more traditional exponential models, the distribution of individual frequencies in an evolving parabolic population is not described by the Maximum Entropy (MaxEnt) Principle in its traditional form, whereby the distribution with the maximum Shannon entropy is chosen among all the distributions that are possible under the given constraints. We sought to identify a more general form of the MaxEnt principle that would be applicable to parabolic growth.

**Results:**

We consider a model of a population that reproduces according to the parabolic growth law and show that the frequencies of individuals in the population minimize the Tsallis relative entropy (non-additive information gain) at each time moment. Next, we consider a model of a parabolically growing population that maintains a constant total size and provide an “implicit” solution for this system. We show that in this case, the frequencies of the individuals in the population also minimize the Tsallis information gain at each moment of the ‘internal time” of the population.

**Conclusions:**

The results of this analysis show that the general MaxEnt principle is the underlying law for the evolution of a broad class of replicator systems including not only exponential but also parabolic and hyperbolic systems. The choice of the appropriate entropy (information) function depends on the growth dynamics of a particular class of systems. The Tsallis entropy is non-additive for independent subsystems, i.e. the information on the subsystems is insufficient to describe the system as a whole. In the context of prebiotic evolution, this “non-reductionist” nature of parabolic replicator systems might reflect the importance of group selection and competition between ensembles of cooperating replicators.

**Reviewers:**

This article was reviewed by Viswanadham Sridhara (nominated by Claus Wilke), Puushottam Dixit (nominated by Sergei Maslov), and Nick Grishin. For the complete reviews, see the Reviewers’ Reports section.

## Background

Population heterogeneity is one of the key properties of any evolving biological system. Heterogeneity amounts to the existence of differences between individuals that could be subject to natural selection and drift which can operate only if the population is non-homogeneous. The dynamics of distributions of individuals within heterogeneous populations and some more complex systems with selection can be described by replicator equations (RE) which capture the ‘basic tenet of Darwinism” [1,2].

A very high or even infinite system dimensionality is one of the principal difficulties in the study of replicator equations. An effective method for solving a wide class of RE based on the reduction theorem has been recently developed and applied to some well-known and new problems concerning the dynamics of heterogeneous populations and communities [3,4].

If the “free” growth of a population is exponential, then the solutions to the corresponding REs have a general property: they minimize the Shannon information gain at each point of the system’s evolutionary trajectory [5]. Hence, the well-known principle of Maximum relative entropy, MaxEnt, which is equivalent to Minimum Information gain [6,7], and is the underlying law for evolving replicator systems.

Szathmary and Maynard Smith [8] represented the model of prebiological evolution of replicators by the equation for the concentration of molecules *dx/dt*= *kx*^*q*^ (hereinafter SS-model). Three cases are distinguished: the exponential case with *q*=1; the super-exponential case with *q*>1; and the sub-exponential case with *q*<1. The models imply “differential survival of the fittest”, “survival of the common”, and “survival of everybody”, respectively [9]. Well established examples of non-exponential population growth apply to global demography (super-exponential or hyperbolic case; *q*=2 [10]) and some molecular replicator systems (sub-exponential or parabolic case; *q*=1/2 [11]).

In fact, the populations of almost all experimentally studied artificial replicators (typically, oligonucleotides that replicate in vitro via binary ligation) grow under the parabolic law [11-13]. The principal cause of the sub-exponential, parabolic growth appears to be product inhibition which slows down the reproduction process compared to the exponential case [14]. Under parabolic growth, dynamic coexistence of competing replicators (survival of everyone) that precludes the action of natural selection is observed under a broad range of parameters [15-17]. However, under certain conditions, in particular, when exponential decay of the replicators is included into the model and/or spatial structures is incorporated, e.g. by allowing the replicators to spread on a surface, selection appears to be possible even under parabolic growth [15,16,18,19].

Thus, parabolic growth appears to be an essential feature of evolving populations of replicators that could be even more directly relevant for biological and prebiological evolution than the exponential growth case. Therefore, understanding the laws governing this type of growth is of potential interest for evolutionary studies. Here we show that for the parabolic growth case, the frequency distribution of the individuals (genotypes) in the population minimizes the Tsallis relative entropy (non-additive information gain) [20] at each time moment, analogous to the maximization of Shannon entropy in the classical, exponential case.

## Results and discussion

In what follows we consider the model of a population composed of distinct individuals (replicators, genotypes or sequences) and described by the SS-models; the dynamics of the replication of each type of individuals is given by the equation:

(1) $$\frac{dx_{i}}{\mathrm{dt}}=k_{i}x_{i}^{q}.$$

We show that the frequency distribution of individual types in the population (1) minimizes the Tsallis relative entropy (non-additive information gain) [20-22] at each time moment. Next, we study the model of a parabolic population that was originally developed by Szathmary & Gladkih [17] (hereafter SG-model) that includes efflux, keeping the total population size constant:

(2) $$\frac{dy_{i}}{\mathrm{dt}}=y_{i}\left(k_{i}y_{i}^{q-1}-\sum_{j}k_{j}y_{j}^{q}\right).$$

Varga and Szathmary [23] demonstrated that the system (2) has a single internal, globally stable rest point with *q*<1. This stable rest point corresponds to the “survival of everybody”, in contrast to the Darwinian case where survival of the fittest prevails, which is realized in standard exponential models with *q*=1. We give a constructive algorithm of solving of system (2). The theorem of Varga and Szathmary immediately follows from this solution. We further show that the frequency distribution of individual types in the population (2) minimizes the Tsallis relative entropy at each moment of the “internal” time of the population.

### Population of freely growing parabolic replicators

The dynamics of the size of a “freely growing” population is given by equation (1). The solution to this equation is

(3) $$\begin{array}{c} x_{i}\left(t\right)={\left(x_{i}{\left(0\right)}^{1-q}+k_{i}t\left(1-q\right)\right)}^{\frac{1}{1-q}}\\ \;=x_{i}\left(0\right){\left(1+x_{i}{\left(0\right)}^{q-1}k_{i}t\left(1-q\right)\right)}^{\frac{1}{1-q}}. \end{array}$$

It can be conveniently written in the form

*x*_*i*_(*t*) = *x*_*i*_(0)*exp*_*q*_(*x*_*i*_(0)^*q*−1^*k*_*i*_*t*)

where $\mathrm{ex}p_{q}\left(x\right)\equiv {\left(1+\left(1-q\right)x\right)}^{\frac{1}{1-q}}$ is the *q*-exponential function. Its inverse is given by the *q*-logarithm function $ln_{q}x=\frac{x^{1-q}-1}{1-q}$. These two functions tend to ordinary exponential and logarithm functions, respectively, as *q* → 1 (see, e.g., [20], ch.3), for formulas and properties of the so-called *q*-calculus).

The total population size is given by the formula

$N\left(t\right)=\Sigma_{i}x_{i}\left(t\right)=N\left(0\right)\Sigma_{i}{\left(P_{0}{\left(i\right)}^{1-q}+k_{i}N{\left(0\right)}^{q-1}\left(1-q\right)t\right)}^{\frac{1}{1-q}}$

From now on we assume for simplicity that *N*(0)=1. Then the frequency of *i*-th individual is

(4) $$P_{t}\left(i\right)=\frac{x_{i}\left(t\right)}{N\left(t\right)}=\frac{{\left(P_{0}{\left(i\right)}^{1-q}+k_{i}\left(1-q\right)t\right)}^{\frac{1}{1-q}}}{\Sigma_{j}{\left(P_{0}{\left(j\right)}^{1-q}+k_{j}\left(1-q\right)t\right)}^{\frac{1}{1-q}}}.$$

**Remark.** It is evident now that

(5) $$P_{t}\left(i\right)=\frac{k_{i}^{\frac{1}{1-q}}}{{\text{Σ}}_{j}k_{j}^{\frac{1}{1-q}}}\;\mathrm{as}\;t\to \infty .$$

This formula reflects the survival of everyone: the frequencies of freely growing “parabolic” replicators, which compose the evolving population, tend to a unique stable state, and each individual (clone) persists and has a non-zero frequency in the limit state of the population. We emphasize that in model (1) there is no interaction between the individuals, and the growth of an individual is bounded neither by its own density nor by the size of the entire population or environment. Formula (4) shows that individual frequencies follow the Pareto distribution at each time moment. This distribution appears as a generalized canonical distribution in non-extensive statistical physics and non-classical information theory [20].

### Dynamical principles of minimal information gain

Classical information theory uses the Boltzmann-Gibbs entropy which is equivalent to Shannon information:

$$S_{\mathrm{BG}}=-\sum_{i=1}^{n}p_{i}\log\;p_{i}$$

Here {*p*_*i*_} is the probability distribution of a full set of *n* events. Information theory developed by Shannon and his successors focused on entropy as a measure of uncertainty of subjective choice. Accordingly, the Principle of Maximum Entropy (MaxEnt principle) is based on the hypothesis that subject to precisely stated prior data, the probability distribution that best represents the current state of knowledge is the distribution with the maximum entropy [6,7,24,25]. The relative Boltzmann-Gibbs entropy was defined by Kullback and Liebler as the divergence between the current distribution *p* and a reference distribution *r* as:

(6) $$D_{\mathrm{KL}}\left(p:r\right)=\sum_{i=1}^{n}p\left(i\right)\log\frac{p\left(i\right)}{r\left(i\right)}$$

Statistical mechanics can be constructed based on the principle of minimum KL-divergence, or information gain, known as the Principle of Minimum Cross-Entropy (MinxEnt) [7]. Recently, it has been shown that within the framework of classical replicator dynamics, the MinxEnt principle is a rigorous mathematical assertion that precisely describes the replicator dynamics [3,5].

The distribution that provides the minimum for the relative BG entropy (KL-divergence) is the Boltzmann distribution that belongs to the family of exponential distributions. The instantaneous distribution of parabolic replicators within a population is not exponential but rather is a power-law distribution (4). Thus, the BG entropy or its variants do not apply to this case. Therefore we ask: can we consider the evolution of such a parabolically growing population similarly to the evolution of an exponentially growing population under an appropriate version of the MinxEnt principle?

The answer to this question is in the affirmative. The Shannon information is not by any account the only possible information measure: a great variety of functions potentially can be useful to measure the missing information in different systems. Many new definitions of entropy and information measures have been invented. Typically, these functions are general entropy measures that include the BG entropy (Shannon information) as a special case [26]. This rich choice begs the question, which information measure is best for a given application.

We submit that the information measure for dynamical models and systems should be chosen in accordance with the system dynamics. In the case of parabolically growing populations, the distribution of the individual frequencies is the Tsallis distribution at each time moment, and accordingly, the Tsallis *q*-entropy is the appropriate information measure. The Tsallis entropy is one of the best known and most widely used among the generalized entropy definitions, and is the basis of non-extensive statistical mechanics [20]. The Tsallis relative *q*-entropy (information gain) of a discrete probability distribution {*p*(*i*)} given a reference distribution {*r*(*i*)} is defined as:

(7) $$\begin{array}{c} I_{q}\left[p:r\right]=\frac{1}{q-1}\Sigma_{i}p\left(i\right)\left({\left(\frac{p\left(i\right)}{r\left(i\right)}\right)}^{q-1}-1\right)\\ =-\Sigma_{i}p\left(i\right)\mathrm{lo}g_{q}\left(\frac{r\left(i\right)}{p\left(i\right)}\right). \end{array}$$

It is also known as the generalized Kullback–Leibler information gain or generalized cross-entropy (see Refs [20-22] for definition, general properties and theorems). The distribution that provides the minimum of the Tsallis information gain (7) with respect to the constraint

(8) $${\text{Σ}}_{i}u\left(i\right)p{\left(i\right)}^{q}=<u{>}_{q}$$

is the distribution

(9) $$\begin{array}{c} p\left(i\right)=\frac{1}{Z}{\left[r{\left(i\right)}^{1-q}-\left(1-q\right)\mathrm{\beta u}\left(i\right)\right]}^{\frac{1}{1-q}}\\ =\frac{r\left(i\right)}{Z}\exp_{q}\left(-r{\left(i\right)}^{q-1}u(i)\beta \right). \end{array}$$

Here Z is the normalization factor (the “partition function”):

$$\begin{array}{c} Z=\Sigma_{i}{\left[r{\left(i\right)}^{1-q}-\left(1-q\right)\mathrm{\beta u}\left(i\right)\right]}^{\frac{1}{1-q}}\\ =\Sigma_{i}r\left(i\right)\exp_{q}\left(-r{\left(i\right)}^{q-1}u(i)\beta \right). \end{array}$$

The Lagrange *(i)* multiplier *β* at a given constraint <*u*>_*q*_ can be found from the equation

(10) $$\frac{\partial }{\partial \beta }\ln_{q}Z=-<u{>}_{q}.$$

where $ln_{q}x=\frac{x^{1-q}-1}{1-q}$ and so $\frac{\partial }{\partial \beta }ln_{q}Z=Z^{-q}\frac{\partial }{\partial \beta }Z$.

One can then calculate the minimum information gain as:

(11) $$\begin{array}{c} I_{q}\left[p:r\right]=-ln_{q}Z-\beta <u{>}_{q}\\ \;=-ln_{q}Z+\beta \frac{\partial }{\partial \beta }ln_{q}Z. \end{array}$$

We can see that the distribution (9) exactly coincides with the distribution (4) of individuals in the population (1):

(12) $$\begin{array}{c} P_{t}\left(i\right)=\frac{{\left(P_{0}{\left(i\right)}^{1-q}+k_{i}\left(1-q\right)t\right)}^{\frac{1}{1-q}}}{\Sigma_{j}{\left(P_{0}{\left(i\right)}^{1-q}+k_{j}\left(1-q\right)t\right)}^{\frac{1}{1-q}}}\\ =\frac{P_{0}\left(i\right)\mathrm{ex}p_{q}\left(P_{0}{\left(i\right)}^{q-1}k_{i}t\right)}{\Sigma_{j}P_{0}\left(i\right)\mathrm{ex}p_{q}\left(P_{0}{\left(i\right)}^{q-1}k_{j}t\right)}. \end{array}$$

when *r*(*i*)=*P*_0_(*i*), *u*(*i*) = *k*_*i*_, −*β*=*t*.

Let us reformulate the above results using “inverse logic”. We do not seek an unknown distribution that would minimize the relative Tsallis entropy subject to a particular set of constraints. Instead, we have the solution (3) of model (1) which produces the distribution (4) at each time moment. Having this distribution, we can compute at each moment *t* the *q*-mean of the reproduction rate, ${\text{Σ}}_{i}k_{i}P_{t}{\left(i\right)}^{q}\equiv <k{>}_{q}^{t}$. Importantly, one can compute this value knowing only the initial distribution *P*_0_(*i*), using the formula:

(13) $$\begin{array}{l} <k{>}_{q}^{t}={\text{Σ}}_{i}k_{i}{\left(\frac{P_{0}\left(i\right)\mathrm{ex}p_{q}\left(P_{0}{\left(i\right)}^{q-1}k_{i}t\right)}{\Sigma_{j}P_{0}\left(i\right)\mathrm{ex}p_{q}\left(P_{0}{\left(i\right)}^{q-1}k_{j}t\right)}\right)}^{q}\\ \;=\frac{\partial }{\partial t}ln_{q}Z\left(t\right). \end{array}$$

where $Z\left(t\right)=\Sigma_{j}{\left[P_{0}{\left(i\right)}^{1-q}+\left(1-q\right)k_{j}t\right]}^{\frac{1}{1-q}}=\Sigma_{j}P_{0}\left(j\right)\mathrm{ex}p_{q}\left(P_{0}{\left(j\right)}^{q-1}k_{j}t\right)$.

The distribution (12) coincides with the distribution which minimizes the Tsallis information gain subject to the constraint (13). Hence, the following theorem holds:

### Theorem 1

*Distribution of parabolically replicating individuals (1) in a population provides the minimum of the Tsallis information gain I*_*q*_[*P*_*t*_:*P*_*0*_] *at each time moment t among all probability distributions that are compatible with the constraint prescribing the current q-mean of the population growth rate,* <*k*> _*q*_^*t*^*.*

The information gain *I*_*q*_[*P*_*t*_:*P*_*0*_] can be calculated as

(14) $$\begin{array}{c} I_{q}\left[P_{t}:P_{0}\right]=-ln_{q}Z\left(t\right)+t<k{>}_{q}^{t}\\ \;=-ln_{q}Z\left(t\right)+t\frac{\partial }{\partial t}ln_{q}Z\left(t\right). \end{array}$$

**Figure 1:**
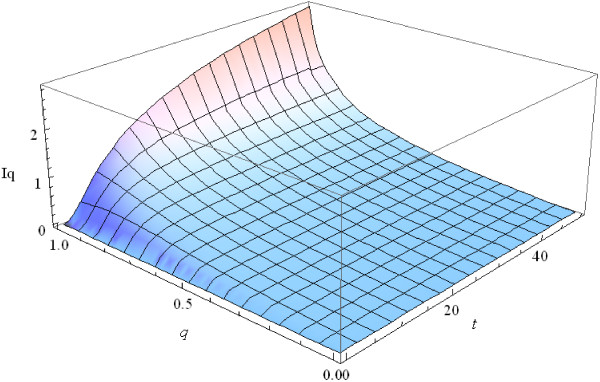
**Dynamics of the Tsallis information gain at different values of *q.***

Figure 1 shows the dynamics of the Tsallis information gain at different values of the parameter *q* when the initials distribution *P*_0_ is uniform.

**Remark**. The difference between the sign of *β* in the distribution (9) and the sign of *t* in the distribution (12) has an obvious explanation. Indeed, in thermodynamics, the frequency of a state decreases with its energy level, whereas in biological populations the fraction of individuals with a higher value of the reproduction coefficient increases with time.

### Population of parabolic replicators with a constant total size and the principles of minimal information gain

Consider now the SG-model [17] of a parabolically growing populations of replicators, with a constant total population size:

(15) $${\dot{y}}_{i}=y_{i}\left(k_{i}y_{j}^{q-1}-\Sigma_{j}k_{j}y_{j}^{q}\right).$$

Without loss of generality, we can assume that *Σ*_*i*_*y*_*i*_(0) = 1.

Equation (15) is a non-linear, high-dimensionality system of ODEs, and its analysis is a non-trivial problem. Varga and Szathmary [23] found an appropriate Liapunov function and demonstrated that the system (15) has a single internal, globally stable rest point. The following Theorem 2 gives an “implicit” solution to system (15) of an arbitrary dimensionality.

Define the deformed moment generating function (*q*-mgf) as:

$M_{q}\left(\delta \right)=\Sigma_{i}\mathrm{ex}p_{q}\left(\delta x_{i}^{q-1}\left(0\right)k_{i}\right)P\left(0,i\right)$

### Theorem 2

*The solution to the population model (15) is given by the formula*

*y*_*i*_(*t*) = *y*_*i*_(0)*exp*_*q*_(*y*_*i*_(0)^*q*−1^*k*_*i*_*τ*(*t*))/*M*_*q*_(*τ*(*t*))

*where τ*(*t*) *is the solution to the Cauchy problem*

(16) dτ/dt=Mqτ1−q,τ0=0.

The theorem reduces the high-dimensionality system (15) to a single equation (16) for the “internal time” *τ* and suggests the following algorithm for solving system (15):

1) Take the solution of equation (1):

$x_{i}\left(\tau \right)={\left(x_{i}{\left(0\right)}^{1-q}+k_{i}\tau \left(1-q\right)\right)}^{\frac{1}{1-q}}=x_{i}\left(0\right)\mathrm{ex}p_{q}\left(x_{i}{\left(0\right)}^{q-1}k_{i}\tau \right)$;

2) Given the initial distribution *P*(0,*i*), compute the function $M_{q}\left(\delta \right)=\Sigma_{i}\mathrm{ex}p_{q}\left(\delta x_{i}^{q-1}\left(0\right)k_{i}\right)P\left(0,i\right)$;

3) Solve the Cauchy problem

*dτ*/*dt* = (*M*_*q*_(*τ*))^1 − *q*^, *τ*(0) = 0;

4) The solution *y*_*i*_(*t*) to problem (15) is given by the formula

*y*_*i*_(*t*) = *x*_*i*_(*τ*(*t*))/*M*_*q*_(*τ*(*t*)).

Theorem 2 immediately implies the Theorem of Varga and Shazmary [23] which we formulate as follows:

**Corollary**. limt→∞yit=ki11−qΣjkj11−q.

(see Methods for the proof of Theorem 2 and the Corollary).

Theorem 2 reduces the model of Szathmary-Gladkih to the free growing parabolic population model (1), so that keeping a constant population size (2) results in convergence of the trajectories to the same equilibrium. In particular, for the Von Kiedrowski’s model with *q*=1/2, we have $\mathrm{li}m_{t\to \infty }y_{i}\left(t\right)\sim k_{i}^{2}$.

**Figure 2:**
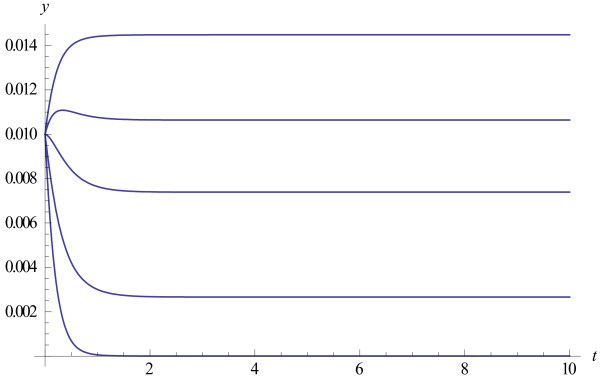
**Evolution of the frequencies of individuals with different growth rates in parabolically growing population.** The results are for *n*=100 and growth rates $k_{i}=\frac{i}{100}$; the three curves correspond to *i*=50, 60, 70 (from bottom to top).

**Example.** A population of “parabolic” replicators is described by the equation (2) with *q*=1/2. The formulas for the solution to this model are derived in the Methods. The plots of the solutions to the model, for the case when the population consists of *n*=100 individuals and the initial distribution is uniform, *y*_*i*_(0)=0.01 for all *i*, depending on the growth rate are shown in Figure 2.

**Figure 3:**
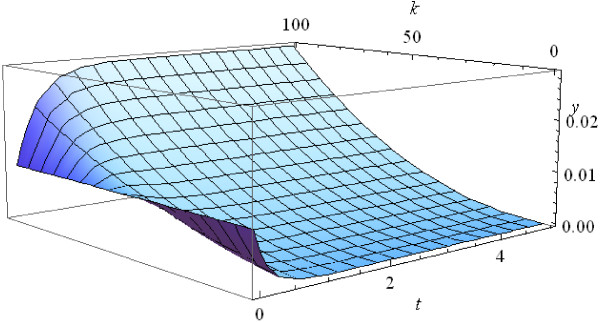
**Evolution of the initial uniform frequency distribution at *n *= 100.**

**Figure 4:**
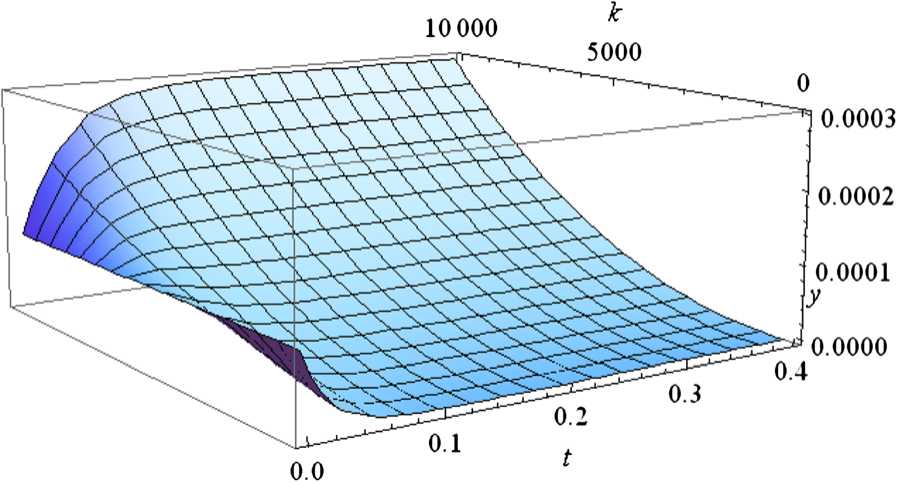
**Evolution of the initial uniform frequency distribution at *n *= 10000.**

Using equation (A3) in Methods, we can trace the evolution of the initial uniform distribution (Figure 3). The population quickly stabilizes and approaches the equilibrium distribution for *t*~4. Note that the larger the size of a population, the faster it approaches the final equilibrium distribution (Figure 3). The plots in Figures 3 and 4 are similar in shape but the population with *n*=10,000 approaches the final distribution much faster, at *t*~0.4.

### Remarks

**Figure 5:**
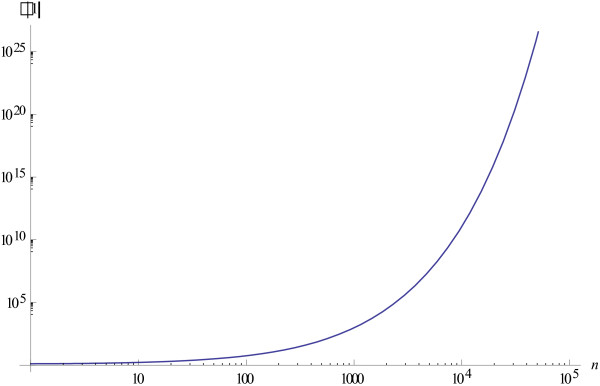
**Internal time τ(1) at the moment of real time equal to 1 against the number of individuals *n *in the population.**

1) *τ*(*t*) increases much faster than *t* because $\frac{d^{2}\tau }{dt^{2}}>0$, hence *y*_*i*_(*t*) = *z*_*i*_(*τ*(*t*)) approaches the limit values very fast (see Example 1 and Figures 2 and 3). Figure 5 shows the values of the internal time *τ*(1) at the moment of real time equal to 1 against the number of individuals *n* in the population.

Overall, the dynamics of the SG- model of a parabolic population coincides with the dynamics of the SS- model for a free growing parabolic population up to replacing the “real time” *t* with the “internal time” *τ* . Taking into account that *τ*(*t*) is a monotonic function of *t* and *τ*(*t*) → *∞* as *t* → *∞*, we conclude that the asymptotic behaviors of both models coincide. For the same reason, the following version of the MinxEnt principle holds for the SG parabolic population.

Let $P_{t}^{*}\left(i\right)=y_{i}\left(t\right)/\Sigma_{j}y_{j}\left(t\right)$ be the current distribution of populations in the SG-model (2). By definition of yit,Pt*i=xiτtΣjxjτt=Pτti where *P*_*τ*_(*i*) is the current distribution of model (1), so

(17) $$P_{t}^{*}\left(i\right)=\frac{P_{0}\left(i\right)\mathrm{ex}p_{q}\left(P_{0}{\left(i\right)}^{q-1}k_{i}\tau \left(t\right)\right)}{\Sigma_{j}P_{0}\left(j\right)\mathrm{ex}p_{q}\left(P_{0}{\left(j\right)}^{q-1}k_{j}\tau \left(t\right)\right)}.$$

The following theorem directly follows from Theorem 1:

### Theorem 3

#### The Principle of minimum of non-extensive information gain

*Distribution P*_t_^*^(17) of the parabolic populations under the SG model provides the minimum of the Tsallis information gain$I_{q}\left[P_{t}^{*}:P_{0}\right]$*at every time moment t among all probability distributions compatible with the constraint prescribing the current q-mean of the individual growth rates,*$\underset{i}{\sum }k_{i}P_{t}^{*}{\left(i\right)}^{q}$*, which is equal to the q-mean growth rate of a population of free-growing replicators at the moment τ(t),*$\underset{i}{\sum }k_{i}P_{t}^{*}{\left(i\right)}^{q}=\underset{i}{\sum }k_{i}P_{\tau \left(t\right)}{}^{q}=<k{>}_{q}^{\tau \left(t\right)}$*.*

There are many other entropy functionals which also may result in different observed distributions. The rich choice from the family of non-classical entropies seems to imply the MaxEnt “anarchism” which was criticized many times as a “senseless fitting” [27,28]. As emphasized above, the main justification for using q-entropies instead of the Gibbs-Boltzmann-Shannon entropy is that the distribution of the variable of interest does not belong to exponential family but belongs to the Pareto distribution family.

An important statement was formulated by Zanette and Montemurro [29]: For any given distribution *p*(*x*), introducing the appropriate function as a constraint <*φ* >_*q*_ exactly yields the distribution *p*(*x*) which provides maximum to the Tsallis q-entropy. In particular, maximization of the Shannon entropy under the constraint <*φ* > where *φ* (*x*) = *Alnp*(*x*)+ *φ*_0_ yields the distribution *p*(*x*). Here the constants *A* and *φ*_0_ fix the origin and units of measure for the average.

Simply put, the result of Zanette and Montemurro [29] states that *any* distribution can be obtained by maximization of any *q*-entropy under the appropriate constraint. Hence, the problem of choosing a particular *q*-entropy (including the Shannon entropy) is reduced to the choice of the “most natural” constraints for the system under consideration [24]. What constraint should be imposed in order to derive the Tsallis distribution by maximization of the Shannon entropy? Let us consider this problem on the example of distribution (4),

$$P_{t}\left(i\right)=\frac{{\left(P_{0}{\left(i\right)}^{1-q}+k_{i}\left(1-q\right)t\right)}^{\frac{1}{1-q}}}{Z\left(t\right)}.$$

Following Zanette and Montemurro,

*φ*_*t*_(*i*) = *A ln*[*P*_0_(*i*)^1 − *q*^ + *k*_*i*_(1 − *q*)t], *A = const*, and the constraint is equal to the prescribed mean value of the function *φ*_t_(*i*),

(18) $$<\varphi_{t}{>}^{t}\equiv \Sigma_{i}P_{t}\left(i\right)\varphi_{t}\left(i\right)=A\Sigma_{i}P_{t}\left(i\right)\ln\;\left[P_{0}{\left(i\right)}^{1-q}+k_{i}\left(1-q\right)t\right].$$

On the other hand, we can obtain the same distribution (4) by maximization the Tsallis entropy under the constraint equal to the prescribed *q*-mean value of the growth rate

(19) $$<k{>}_{q}^{t}=\Sigma_{i}k_{i}P_{t}{\left(i\right)}^{q}.$$

The mean value of the growth rate is a natural, biologically relevant quantity as opposed to the constraint (18) which has no natural interpretation. That is why we favored the Principle of minimum of Tsallis information gain against the Shannon information gain when we deal with the Pareto distribution.

## Conclusions

It follows from Theorems 1 and 3 that Tsallis entropy is the adequate information measure for the distribution of individual frequencies in the SS and SG models of population evolution with parabolic growth. The quantities *I*_*q*_[*P*_*t*_:*P*_0_] and *I*_*q*_[*P*_*τ*(*t*)_:*P*_0_] represent the information gain in the population up to the moment *t* in the SS and SG models, respectively. The Tsallis entropy and distribution include the standard Shannon entropy and the Boltzmann-Gibbs distribution as a special case when *q*→1.

A fundamental property of the Tsallis entropy is that it is non-additive for independent subsystems: *I*_*q*_[*S*^(1)^ * *S*^(2)^] = *I*_*q*_[*S*^(1)^] + *I*_*q*_[*S*^(2)^] + (1 − *q*)*I*_*q*_[*S*^(1)^]*I*_*q*_[*S*^(2)^], where *S*^(1)^,*S*^(2)^ are two independent partitions of the complete system *S*.

The entropy index *q* characterizes the degree of non-additivity. Thus, for parabolic systems with *q <* 1 such as typical systems of molecular replicators, the information about two exhaustive independent subsystems is insufficient to obtain the information about system as a whole (the opposite is true for hyperbolic replicator systems with *q* > 1 that contain less information than the sum of the information contents for independent parts). In the above expression, the term (1−*q*)*I*_*q*_[*S*^(1)^]*I*_*q*_[*S*^(2)^] may be considered an interaction term. With respect to prebiotic evolution, this “non-reductionist” character of parabolic replicator systems might reflect the importance of the interaction between genetic elements that could encode complementary functions and form ensembles of “selfish cooperators” subject to group selection [30,31].

On a more general note, the results of this analysis indicate that the MaxEnt (MinxEnt) principle is a general optimization principle that governs the evolution of populations of replicators regardless of the specifics of the growth dynamics. Only the choice of the appropriate entropy (information) function depends on the growth law of a particular class of systems.

## Methods

Proof of Theorem 2.

Let us consider the equation

(A1) $$dx_{i}\left(\tau \right)/\mathrm{d\tau}=k_{i}x_{i}^{q}\left(\tau \right),x_{i}\left(0\right)=y_{i}\left(0\right),$$

which coincides with (1) up to the notation of independent variable; the “internal time” *τ* will be defined later.

Define the frequencies *z*_*i*_(*τ*) = *x*_*i*_(*τ*)/*N*(*τ*) where *N*(*τ*) = *Σ*_*i*_*x*_*i*_(*τ*). Then

$$\begin{array}{c} dz_{i}/\mathrm{d\tau}=k_{i}x_{i}^{q}/N-\frac{x_{i}}{N^{2}}\Sigma_{j}k_{j}x_{j}^{q}\\ \;=N^{q-1}z_{i}\left(k_{i}z_{i}^{q-1}-\Sigma_{j}k_{j}z_{j}^{q}\right). \end{array}$$

Let us recall that the solution to (A1) is known,

$$\begin{array}{c} x_{i}\left(\tau \right)={\left(x_{i}{\left(0\right)}^{1-q}+k_{i}\tau \left(1-q\right)\right)}^{\frac{1}{1-q}}\\ =x_{i}\left(0\right)\mathrm{ex}p_{q}\left(x_{i}{\left(0\right)}^{q‒1}k_{i}\tau \right) \end{array}$$

and hence *N*(*τ*) and z_*i*_(*τ*) are also known.

Given the initial values *x*_*i*_(0), define the function

$$M_{q}\left(\delta \right)=\Sigma_{i}\mathrm{ex}p_{q}\left(\delta x_{i}^{q-1}\left(0\right)k_{i}\right)x_{i}\left(0\right).$$

Let us emphasize that *M*_*q*_(*δ*) is a well determined function as the initial values {*x*_*i*_(0)} are known. The current community size for model (A1) is then

(A2) $$N\left(\tau \right)=M_{q}\left(\tau \right).$$

and

(A3) $$z_{i}\left(\tau \right)=\frac{x_{i}\left(\tau \right)}{N\left(\tau \right)}=\frac{\mathrm{ex}p_{q}\left(x_{i}^{q-1}\left(0\right)k_{i}\tau \right)}{\mathrm{Mq}\left(\tau \right)}x_{i}\left(0\right).$$

The “internal time” *τ* (*t*) was defined as the solution to the Cauchy problem (16)

$$\mathrm{d\tau}/\mathrm{dt}=M_{q}{\left(\tau \right)}^{1-q},\tau \left(0\right)=0.$$

Define *y*_*i*_(*t*) by the formula *y*_*i*_(*t*) = *z*_*i*_(*τ*(*t*)); then {*y*_*i*_(*t*)} solve the system (15):

$$\begin{array}{c} \frac{dy_{i}\left(t\right)}{\mathrm{dt}}=\frac{dz_{i}}{\mathrm{d\tau}}\frac{\mathrm{d\tau}}{\mathrm{dt}}=N^{q-1}\left(t\right)z_{i}\left(\tau \left(t\right)\right)(k_{i}z_{i}^{q-1}\left(\tau \left(t\right)\right)\\ -\Sigma_{j}k_{j}z_{j}^{q}(\tau \left(t\right))N^{1-q}\left(t\right)\\ =y_{i}\left(t\right)\left(k_{i}y_{i}^{q-1}-\Sigma_{j}k_{j}y_{j}^{q}\right). \end{array}$$

The theorem is proven.

**Corollary**. $\lim_{t\to \infty }y_{i}\left(t\right)=\frac{k_{i}^{\frac{1}{1-q}}}{\Sigma_{j}k_{j}^{\frac{1}{1-q}}}.$

It follows from formula (A2) and equation (16) that $\frac{\mathrm{d\tau}}{\mathrm{dt}}=N{\left(\tau \right)}^{1-q}$. Equations (A1) implies that $\frac{\mathrm{dN}\left(\tau \right)}{\mathrm{d\tau}}>0,$ so *N*(*τ*) is a monotonically increasing function of *τ* and hence *τ*(*t*) → ∞ monotonically as *τ* increase. Next, *y*_*i*_(*t*) = *z*_*i*_(*τ*(*t*)) = *x*_*i*_(*τ*(*t*))/*N*(*τ*(*t*)), so

$\mathrm{li}m_{t\to \infty }y_{i}\left(t\right)=\mathrm{li}m_{\tau \to \infty }\frac{x_{i}\left(\tau \right)}{N\left(\tau \right)}=\frac{k_{i}^{\frac{1}{1-q}}}{\Sigma_{j}k_{j}^{\frac{1}{1-q}}}$ according to formula (5).

## Reviewers’ reports

### Reviewer 1: Viswanadham Sridhara (nominated by Claus Wilke, University of Texas, Austin)

In this manuscript, the authors studied the distribution of frequencies of individuals (genotypes) in parabolic (sub-exponential) population growth. They claimed that this distribution of individual frequencies follow the Pareto law and minimize the Tsallis information gain, in contrast to minimization of Shannon information gain for exponential population growth models, although it is to be noted that one variant of Tsallis distribution gives rise to Boltzmann-Gibbs distribution (i.e., as q->1). The authors used previously published models on parabolic population growth (SS [8], SG [17]) in combination with their methods to verify their proposed claims.

The authors were able to show that, indeed such distribution of individual frequencies minimize Tsallis information gain. This work is a good extension to the previously published work by the same authors on solving Replicator Equations (Karev et. al. [3,4]). In summary, minimum information gain is hence shown to be the underlying law for sub-exponential, exponential and super-exponential population growths.

I have no specific requests for changes.

### Reviewer 2: Puushottam Dixit (nominated by Sergei Maslov. Brookhaven National Laboratory)

In this work, the authors generalize their previous result on the relationship between the Gibbs-Boltzmann-Shannon entropy and the exponential growth replicator equation [3] by analyzing parabolic and hyperbolic growth models. They show that the frequency distribution of species growing with a modified exponential dynamics is best described by a Tsallis q-exponential distribution. I find the mathematical results of the work interesting but I think that the physical conclusions are not clearly delineated. I would like the authors to considerably extend their discussion about the biological implications of their results before I can recommend the article to be published in Biology Direct.

Response: *we certainly realize the value of biological implications. However, this paper primarily aims at presenting mathematical/information-theoretical results that apply to a biologically most realistic replicator system, that is a parabolically growing one. Hence the biological relevance. We do discuss what we think is an interesting biological implication, namely the applicability of this non-additive formalism to cooperative behavior of prebiotic replicators; this part was reworded in the revision to clarify. We tend to believe that further biological speculation* would *be excessive at this stage*.

My specific questions are below.

In the current work, the connection between system dynamics and the information theoretic quantities such as entropy and mutual information (either Gibbs-Shannon or Tsallis) arises solely because the solution of the growth equation takes a certain form (either exponential or q-exponential). Apart from serving as a quantifier of the variability, in a deterministically growing population the connection between the entropy/mutual information computed here and the notion of belief/probability is not clear (after all, we are talking about a completely deterministic process).

Response: *Yes, both Szathmary-Smith and Szhathmary-Gladkih models are completely deterministic. Having a solution of these multi-dimensional processes, x*_*i*_*(t), we can define the frequencies of different species, x*_*i*_(*t*)/*N*(*t*)*, where N*(*t*) *is the total population size. Then, it is a standard approach to identify the frequencies of species with probabilities (to get an individual from a given species after randomly choosing an individual from the total population). We do not elaborate and even do not use here any connection between the entropy/mutual information and the notion of belief/probability apart from the mathematical definition of the relative q-entropy as a measure of information contained in a given probability distribution.*

I would suggest that the authors replace the somewhat confusing information theoretic terms and adopt something along the lines of ‘population variability’. Or, the authors may provide a justification for using the information theoretic glossary in terms of earlier work in ecology in estimating species frequencies. See for example, Dewar and Porté [32].

Response: *The information theoretical terms used here are standard, so we do not see the necessity to justify them here from first principles; there is a huge literature on the basic concepts of the theory and its applications in different areas including the interesting paper of Dewar and Porté*[32]*as well as the vast body of work by Jaynes and his followers on which we capitalize here*[6,7]*.*

Q-entropies should be used only when there is a good reason to expect non-extensivity in the system. The authors first derive the frequency distribution from a deterministic equation and then show that it can also be obtained by maximizing the Tsallis entropy under suitable constraints. I think this is a fascinating result and I would really like the authors to extend their very short discussion to include a justification of the use *q*-entropies for prebiotic growth instead of the usual Gibbs-Boltzmann entropy. This justification should not invoke the underlying modified exponential dynamics, which lead to the Tsallis entropy in the first place.

Response: *Indeed, the use of q-entropy has to be justified by properties of the system such as non-extensivity (more precisely, non-additivity*[20]*) when it is employed to derive an unknown probability distribution. However, when it is already known that the system is described by power law/Pareto distribution, it follows that these distributions can be obtained from maximization of the Tsallis q-entropy. It is well known (theorems of Jaynes and Kullback) that maximization of the relative Boltzmann-Gibbs-Shannon entropy results in distributions that belong in the exponential family. In other words, the MaxEnt principle in this case is merely a restatement of the fact that the distribution belongs in the exponential family. Similarly, the Principle of maximum of the relative q-entropy is merely a restatement of the fact that the given distribution belongs to the Pareto (or Tsallis) family. Hence, q-entropy and the corresponding variational principle may be used in each case where the Pareto (or Tsallis) distribution is observed; the applicability of these approaches does not depend on the assumption on the non-additivity of the system.*

In other words, assuming that we did not know the equations governing the population dynamics, what unusual properties of the prebiotic world serve as a rationale for using Tsallis entropy (instead of the usual Gibbs-Shannon entropy) to estimate the frequencies of species? Perhaps the answer lies in the following observation by Plastino [33]: A system interacting with a small bath of ideal gas particles (as opposed to a large thermodynamic bath) behaves as if its Tsallis entropy is maximized. In short, Tsallis entropy is a special case of the Gibbs-Shannon entropy if baths are small.

Response: *Actually, as indicated in the present article, the parabolic growth of replicator systems follows from a feature that cannot be considered unusual but is manifest in most chemical systems, namely product inhibition of the reaction, in this case replication*[11,15]*. In more general terms, as emphasized in the literature including the quoted work of Plastino and Plastino*[33]*, non-extensive thermostatistics (NEXT) is based upon the following two postulates:*

In practice, it is difficult to expect that these postulates can be verified directly for different complex systems of interest. In most cases, the validity of the postulates should be decided exclusively on the basis of the conclusions to which they lead and their comparison with experiment. The main point is that the variable of interest in the system follows the Pareto-distribution, and this is the case for models of prebiotic evolution where the frequencies of species follow the Pareto distribution and growth rate is the observable variable.

*Moreover, the frequencies of species have the Pareto distribution* (1+*ax*)^-*b*^*at each time moment with the parameter a proportional to time. We further expand on these issues in the revised discussion.*

*The physical interpretation based on the small thermodynamic bath is certainly of interest and probably worth exploring in future models of prebiotic replicator systems but this is beyond the scope of the present article*[27-29]*.*

1) *The entropy of a system is given by the q-entropy;*

2) *Experimental measurement of an observable variable yields the q-expectation value.*

### Reviewer 3: Nick Grishin, University of Texas Southwestern Medical Center, Dallas

This study elaborates on a known fact that Tsallis distribution originates upon maximization of Tsallis entropy under appropriate constraints and discusses the relevance of this to biological systems. The constraint used is a constant generalized mean (“*q*-mean”) which generates a family of q-Exponential distributions. When *q*=1, regular Shannon entropy, which produces Boltzmann distribution under the constraint of constant mean (i.e. conservation of energy in a system) is a special and well-known case. While mathematical part of the paper is more like a review (e.g. the main results can be seen on Wikipedia pages and papers and books they reference), I have not seen elaboration of these theories using biological systems.

Response: *The aim of the paper is not elaboration of the theory of non-extensive entropies neither its using for derivation of distributions of biological systems. We gave a short review of this theory, but the math part of the paper is devoted mainly to solving of non-exponential models of inhomogeneous populations. Then we gave an interpretation of this solution from the point of view of the Principle of minimum of Tsallis information gain. These math results are new, to the best of our knowledge.*

It would be very interesting if the authors could elaborate on biological meaning of such theories. One obvious property (non-additivity) was mentioned, but what could be a broader picture of maximum Tsallis entropy application to derive evolutionary laws? Is this just a cute trick to obtain phenomenological equation that Szathmary & Smith introduced, or there is more meaning and usefulness to it in deriving some more mechanistic and predictive models? Such discussion could very significantly increase the value of this study.

Response: *We do not derive evolutionary laws from maximization of the Tsallis entropy. We move in the opposite direction: we prove that the distribution of clones in non-exponential population model is the Tsallis (or Pareto-like) distribution. Hence, under appropriate constraint, the system dynamics obeys the Principle of minimum Tsallis relative entropy independently on if we accept (believe in) this Principle or not and independently on any particular properties of the population. Non-additivity of information gain is not a property of the system under consideration postulated a priory, but is the last element in the logical chain:*

non-exponential dynamics -> Tsallis distribution of clones at each moment -> minimum of the Tsallis information gain at each moment -> Tsallis relative entropy as a measure of information gain consistent with the system dynamics -> non-additivity of the information measure.

*There exists a huge literature devoted to the derivation of particular (including experimental) distributions from variational principles. The MaxEnt principle and the Tsallis formalism have been already applied to many problems in widely different areas (physics: astrophysics, cosmology, turbulence phenomena; mathematics: Lèvy flights, superdiffusion, non- linear Fokker-Planck equations, economy: analysis of market trends; biology and medicine, etc.; see some references at*http://tsallis.cat.cbpf.br/biblio.htm*).*

*It seems that the only common property of all these systems is non-additivity of the entropy functional; actually it is a formal mathematical assertion, which follows directly from the axiomatic for the Tsallis entropy (see*[34]*and references therein (for Generalized Shannon-Khinchin axioms).*

With regard to the biological meaning, as pointed out in the manuscript and in our response to reviewer 2, it stems from the fact that Tsallis q-entropy naturally applies to biologically realistic parabolic replicator systems unlike the Shannon-Boltzmann entropy which only applies to idealized exponential systems. We also offer a biological interpretation of the non-additivity of the q-entropy. We believe that at this stage these are the necessary and sufficient biological implications.

On the other hand, I am interested to learn what conditions imposed on the system yield sub- or super-exponential behavior after maximization of Shannon's entropy? The results do not have to exactly match the Szathmary & Smith growth equation, of course, but be qualitatively similar. E.g. maximization of Shannon entropy while keeping the mean constant results in Boltzmann distribution, and when the variance is kept constant, Gaussian distribution emerges. Maybe such conditions, if found, could shed some light on biology and evolution of these systems.

Response: *It is known that maximization of the Shannon's entropy under prescribed geometrical mean results in the Pareto distribution*[35]*. In general, any distribution can be obtained by maximization of the Shannon's entropy under appropriate constraints*[29]*, and hence the problem is in the choice of the constraints that are “most natural” for the system of interest.*

Some more technical issues:

1. It seems that starting background section of the abstract with a sentence that contains two words in quotes and two sets of parenthesis does not help in communication and might turn perspective readers off. It would be better to have a more accessible and friendly background section.

Response: *we removed the quotes that were not strictly necessary in this case. As for the terms in parentheses, they clarify the meaning of the preceding terms and as such, we think, are helpful and hopefully not too annoying.*

2. It would be nice to carefully proofread the text for grammar. I saw quite a few trivial lapsi, e.g. the first sentence in the abstract or “We sought to identifiable” also in the abstract.

Response: *we regret these unfortunate and indeed trivial errors. These were corrected to the best of our ability.*

## Competing interests

The authors declare that they have no competing interests.

## Authors’ contributions

GPK performed the analysis; GPK and EVK interpreted the results and wrote the manuscript. Both authors read and approved the final manuscript.
